# Applications of Silver Nanoparticles in Pediatric Dentistry: An Overview

**DOI:** 10.7759/cureus.26956

**Published:** 2022-07-17

**Authors:** Monika Khubchandani, Nilima R Thosar, Suwarna Dangore-Khasbage, Rashi Srivastava

**Affiliations:** 1 Pediatric Dentistry, Sharad Pawar Dental College and Hospital, Datta Meghe Institute of Medical Sciences, Wardha, IND; 2 Pediatric and Preventive Dentistry, Sharad Pawar Dental College and Hospital, Datta Meghe Institute of Medical Sciences, Wardha, IND; 3 Oral Medicine and Radiology, Sharad Pawar Dental College and Hospital, Datta Meghe Institute of Medical Sciences, Wardha, IND

**Keywords:** caries prevention, antibacterial material, silver nanoparticles, pediatirc dentistry, nanodentistry

## Abstract

Nanoscience and nanotechnology are emerging fields involved in the synthesis and application of nanoscale materials and structures. Metallic nanoparticles and metallic oxide are being used extensively in dentistry as they interfere with bacterial metabolism and prevent biofilm formation. AgNPs are a class of zero-dimensional materials with distinctive morphologies. The metallic nanoparticles demonstrate the significant antimicrobial activity by ion release, oxidative stress induction, and non-oxidative mechanisms. Metallic silver has been known for its antimicrobial activity since ancient times. Through the years, silver-containing compounds have been used in various forms to treat several medical conditions. Incorporating silver nanoparticles into dental materials may enhance the mechanical features and antibacterial properties of dental materials. Therefore, an increasing number of dental materials with the inclusion of silver nanoparticles are being developed that improve the overall oral health status of patients. This paper aims to review the literature on specific characteristics of silver nanoparticles and their applications in pediatric dentistry.

## Introduction and background

Metallic silver is well known for its antimicrobial effect since early times. Through the years, silver-containing compounds have been used in various forms for several medical conditions such as wound healing, burns, and ulcer treatment largely pragmatically before the realization that microorganisms were the infection-causing agents. Among plenty of useful properties, silver has potent antimicrobial action against a wide range of pathogens [1]. In the past few decades, antibiotic-resistant bacteria have been observed with increasing frequency endangering the efficacy of antibiotics; hence, it is imperative to produce effective bactericides so as to control bacterial infections [2].

Silver nanoparticles: The word “Nano” originated from the Greek term “Nanos”, analogous to dwarf or very small. The term applies to particles with a size range of 1nm to 100 nm, containing about 20 to 15,000 silver atoms [3]. Silver nanoparticles can be prepared into different sizes and shapes by a variety of routes, including a physical, chemical, and biological synthesis [4].

Silver nanoparticles have been known for centuries for their extensive biomedical applications like antibacterial, antifungal, and anti-plasmodial. However, the antibacterial effect is dependent on the concentration and rate of ionic silver release. Silver nanoparticles owing to their nano-scale dimensions and potentially large surface area, can directly interact with the bacteria, cause structural changes, and lead to cell death [3].

A silver nanoparticle is known for its antibacterial and anti-adherence properties against streptococcus mutans, which is the main pathogen responsible for dental caries. Silver nanoparticle 1-10 nm size demonstrates significant antibacterial and anti-adherence activity against *S. Mutans* bacteria [5].

There are different types of bacterial species having different membrane structures. The bacteria have been broadly categorized as Gram-positive and Gram-negative. The key features which differentiate between the two are the density of the cell wall and existence of outer lipid membrane. Gram-positive bacteria possess a cell wall consisting of about 30 nm thick peptidoglycan layer but lack the outer lipid membrane. Gram-negative microbes retain a much thinner peptidoglycan layer (∼2-3 nm) which is surrounded by a membrane of lipopolysaccharide [6].

## Review

Mechanisms of antimicrobial action - silver nanoparticles are one of the most widely used nanomaterials in dentistry. The antibacterial effect of nanoparticles is dependent on their size. The smaller particles within 1-10 nm size provide a potent antibacterial effect as they have a greater surface area to contact with microbial cells [7]. The silver ions target multiple sites in the bacterial cell that bring about morphological and structural changes within the bacteria. The mechanism of action of silver nanoparticles is depicted in (Table 1).

**Table 1:** Mechanisms of Antimicrobial Action

Target site	Mechanisms of antimicrobial action
Reaction of Silver nanoparticles with peptidoglycan cell wall	Silver ions being highly reactive, interact, and bind to the negatively charged bacterial cell wall, alter its permeability, and cause cell damage. Gram-negative bacteria constitute a thin layer of peptidoglycan which is covered by an outer cell envelope of lipoprotein, phospholipid, and lipopolysaccharide. The role of the outer lipid layer is to allow selective permeability to certain products. The bacterial membrane is known to contain many sulfur-containing proteins. Since silver has a higher propensity to react with sulfur and phosphorus compounds, biologically active silver ions bind to the outer membrane creating changes in membrane morphology and leading to an increase in membrane permeability. Some studies demonstrate mutual electrostatic attraction between the negatively charged bacterial cell and positively charged silver ions [8]. In gram-positive bacteria, the thick cellular wall may decrease the permeation of silver nanoparticles. The bactericidal effect of silver nanoparticles is dependent on their size and dose. Silver nanoparticles with sizes 1-10 nm show maximum interaction with the bacterial surface [6].
Action on Plasma membrane	Apart from the ability to release silver ions, nanoparticles themselves can lead to cell lysis. Nanoparticles of smaller than 10 nm size are highly reactive owing to the greater surface area of interaction with bacterial cells. [6] These nanosized particles can permeate through the cell membrane, causing structural changes within the bacterial casing, and inhibiting the uptake of phosphate compound, which is one of the essential components for several biological processes within the bacteria such as energy metabolism, membrane integrity, inheritance of genetic materials, and intracellular signaling [9]. Ionic silver interacts with thiol group compounds of respiratory enzymes present within the bacterial cell, thereby affecting cellular respiration and producing reactive oxygen species [6]. Transmission electron microscopy (TEM) revealed that *E. Coli* bacteria treated with silver showed enlargement of periplasmic space suggestive of shrinkage of the inner membrane and its separation from the cell wall. However, *Staphylococcus aureus*, gram-positive bacteria, underwent comparable morphological changes as *E. coli*, howbeit to a smaller extent, having a thicker cell envelope suggesting greater resistance to silver ions [10,11].
Action on cytoplasmic DNA	Ag+ ions are strong nucleic acid binders having an affinity to interact and form complexes with bases present in DNA molecules. The transmission electron microscopic image showed a normal, random distribution of electron-light region assigned as normal DNA. However, following treatment with Ag+ ion, an electron light region appeared in the center of the *E. coli* showing a tightly condensed substance twisted together. [10] Such damage to DNA blocks its replication ability. When DNA molecules are relaxed and distributed in all parts of the cell, cellular replication can effectively take place. But when DNA is twisted in the center of the cell, it is no longer able to continue replication; hence, leading to cell death. [12]

Applications of silver nanoparticles in pediatric dentistry

Silver nanoparticles have been proven as potent antibacterial agents in preventing dental caries in children. Previous research [13] has shown effective anti-cariogenic activity against *Streptococcus mutans* in in-vitro experiments.

Its virulence is 25 times greater than other agents like chlorhexidine. Additionally, it portrays antifungal and antiviral activity. Hence, various preparations containing silver nanoparticles are useful in preventing debilitating conditions such as Early Childhood Caries (ECC) and Rampant Caries. This is mainly due to the ability of the silver nano-particles to invade and breakdown the biofilm matrix [14].

Silver nanofluoride (NSF)

New formulations combining silver nanoparticles and fluoride were recently observed. The silver nanofluoride suspension was tested in-vitro for its efficacy against cariogenic pathogens as well as cytotoxicity. The tested MIC was 33.54 μg/ml, and MBC was 50.32 μg/ml. When compared with Silver diamine fluoride (SDF) the values turned out to be similar. However, in this study, the staining of the enamel surface was not evaluated [14].

A formulation containing silver nanoparticles, fluoride, and a vehicle of chitosan was also evaluated. A single application of this customised varnish was done on dental caries and observed at intervals of seven days, five months and twelve months. At the time period of seven days, 81% of the samples had arrested cavities at the fifth month evaluation, 72.7% of the samples showed evidence of arrested carious lesions, and after 12 months, 66.7% of the carious teeth were still arrested. Dark and black stains were not observed on the enamel of the teeth [15].

Similarly, silver nanoparticles were added to a commercially available fluoride varnish and evaluated for remineralization of primary teeth with white spot lesions. Anterior primary teeth were included in the study after examination using the DIAGNOdent laser wand. A preparation of silver nanoparticle powder and fluoride varnish was prepared at 0.1% wt. One tooth per quadrant was coated with this experimental varnish, and the opposing quadrant was coated with the normal control fluoride varnish. The treatment was carried out once every week for three weeks. Subsequent evaluations were done using the DIAGNOdent at three months to evaluate changes in remineralization. The rate of remineralisation was found to be higher than plain water on treatment with silver nanoparticles [16].

Pit and fissure sealants

In another research, the efficacy of pit and fissure sealants with added silver nanoparticles was evaluated. The microleakage of this experimental sealant was assessed in first permanent molars in comparison with the conventional sealants. It was found that the conventional sealant demonstrated an average microleakage of 30.6%, and the silver nanoparticles mixed sealant showed 33.6%. Additionally, a substantial reduction in fluorescence was seen in the silver nanoparticle sealant group.

The adhesion of the pit and fissure sealants with silver nanoparticles showed an average difference more than 25% between the groups. The silver nanoparticle group had a tendency to diminish fluorescence faster. This suggested that the teeth tended to remineralize when the microorganism caused its demineralization [17].

Glass ionomer cement (GIC) with silver nanoparticles

The poly acrylic acid (PAA), which is a constituent of the conventional GIC, in an aqueous solution, produces polyacrylate anions (PA−) with uncoordinated carboxylate ions (COO−). These have the ability to bind metallic cations such as silver salts (Ag+). In a study evaluating the antimicrobial efficacy and compressive strength of glass ionomer cement with silver nanoparticles, the customized one-step synthesis of nanosilver in a long-chain PAA aqueous solution was described. This was followed by the photoreduction of Ag+ ions to silver nanoparticles. This procedure was hypothesized to impart superior characteristics without disturbing the inherent structure of the cement. The higher concentration of silver in the matrix of this customized cement (NanoAg-GIC) increased the setting time by 32% and displayed significant cell inhibition zones for *Escherichia coli*. This indicated that the Ag+ ions even showed diffusion on the surrounding material. Further, the metabolic activity of *Streptococcus mutans* was also affected by the NanoAg-GIC [18].

Orthodontic applications

Investigations for the assessment of composite resins with silver nanoparticles were carried out. This was done to evaluate the ability of such resins to reduce white spot lesions adjacent to the luted orthodontic brackets. TiO2 nanoparticles were used in the comparative group. A light-cure orthodontic resin was loaded with 0.5% (w/w) nano-silver particles (Ag, 99.99%, 20 nm) through homogeneous mixing of the two materials. This was done to ensure a uniform mixture of the nanoparticles. The brackets were bonded onto the buccal surfaces of the extracted teeth and exposed to the cariogenic model. Overnight cultures of *Streptococcus mutans* (ATCC #35668) and *Lactobacillus casei* (ATCC #39392) in a dextrose-free trypticase soy broth, supplemented with 5% sucrose (TSBS), at 37°C was prepared and used as the inoculum. Three separated locations were tested for demineralization, and while teeth in both the experimental groups had statistically higher hardness indices at two locations, at the third point of assessment, significantly higher hardness indices were calculated for the teeth in the silver group. Even at a distance from the cured resin, the effect of silver nanoparticles was found to be more potent and effective [19].

## Conclusions

The use of silver nanoparticles in the biomedical sciences is quickly progressing due to their potent antimicrobial action as a result of their high volume-to-surface ratio. The incorporation of silver nanoparticles into dental materials provides a great antimicrobial effect. These metallic nanoparticles are known to release silver ion, which inhibits planktonic bacterial cells that attach themselves to the surface and produce biofilm structures. Due to their ability to obstruct microbial colonization, silver nanoparticles are added to various dental materials such as acrylic resins, root canal fillings, glass ionomer cement, pit and fissure sealants accentuating microbial growth inhibition, and physical properties of the modified materials. Although the research on silver nanoparticles incorporated in dental materials has demonstrated favorable results, long-term clinical trials are needed to monitor the clinical effectiveness and overall benefits on patients’ oral health.
